# Which lupus trial endpoints best reflect clinical judgment or biomarker improvement?

**DOI:** 10.1186/ar3981

**Published:** 2012-09-27

**Authors:** A Thanou, M Munroe, S Kamp, F Carthen, JA James, JT Merrill

**Affiliations:** 1 Oklahoma Medical Research Foundation, Oklahoma City, OK, USA

## Background

Outcome measures used in clinical trials of lupus are complex and difficult to interpret. With a plethora of new treatments in development and no objective gold standard to define efficacy, a better understanding of what the different endpoints signify would be helpful in designing more efficient trials and in informing clinicians in practice who need to interpret the results.

## Methods

Ninety-one patients from the Oklahoma Lupus Cohort (five males, mean age 41) were identified with two visits at which SLEDAI and BILAG scoring had been performed and active disease (SLEDAI >6) was present at the first visit. Each was evaluated by physician judgment as the same, improved or worse at the second visit based on clinical records. Serum cytokine levels were measured by xmap multiplex bead-based assay.

## Results

At baseline, the mean (SD) PGA, SLEDAI and BILAG scores were 1.75 (0.37), 10.0 (4.09) and 15.1 (6.54). Sixty-eight patients were ranked as improved, 23 as the same or worse at the follow-up visit. The SLE Responder Index (SRI) and BILAG-based Composite Lupus Assessment (BICLA) were compared. Endpoints using these constructs restrict medication use. SRI and BICLA without medication criteria captured physician-ranked improvement (PRI) with 85.3% versus 76.5% sensitivity and 73.9% versus 78.3% specificity. With medication limits, fewer patients were responders, but specificity increased to 82.6 and 95.6%. Similar trends were observed for modified SRI scores (SRI3 and SRI5). Spearman rank correlations to PRI were: SRI3 = 0.605, SRI4 = 0.563, SRI5 = 0.541, BICLA = 0.492 (all *P *< 0.000001). All nine patients who improved by BICLA but not SRI failed to achieve four-point improvement in the SLEDAI, which requires complete resolution of one or more disease features. However, seven were rated as significantly improved by PRI. All 15 responders to SRI and not BICLA failed to improve in every organ, but 12 were rated as improving by PRI. Biomarkers could provide an objective standard to compare clinical measures. Exploratory evaluation of serum cytokines might allow some preliminary modeling. In the current study IL-6 was only detectable in a minority of patients (*n *= 29) but decreased significantly in those patients who improved: PRI *P *< 0.001, SRI3 and SRI 4 *P *= 0.003, SRI5 *P *= 0.001, BICLA *P *= 0.005.

## Conclusion

SRI5 and BICLA, with the addition of medication restrictions, may be the most specific measures for improvement despite risking loss of sensitivity, and could provide the most meaningful proof of efficacy in an appropriately powered clinical trial. Shortfalls of SRI and BICLA are usually due to the BICLA requiring only partial improvement but in all organs versus SRI requiring full improvement but not necessarily in all organs. Physician's overall opinion corresponds as well as or better than formalized endpoints to improvements of IL-6 in an exploratory biomarker analysis of a lupus patient subset.

